# Association between lifestyle factors and decreased kidney function in older adults: a community-based cross-sectional analysis of the Taipei City elderly health examination database

**DOI:** 10.1186/s12882-020-01838-1

**Published:** 2020-05-08

**Authors:** Horng-Jinh Chang, Kuan-Reng Lin, Meng-Te Lin, Junn-Liang Chang

**Affiliations:** 1 Department of Management Sciences, Tamkang University, No.151, Yingzhuan Rd., Tamsui Dist, New Taipei City, 25137 Taiwan, Republic of China; 2 Department of Pathology & Laboratory Medicine, Taoyuan Armed Forces General Hospital, No.168, Chung-Shing Rd., Long-Tang District, Taoyuan City, 325 Taiwan, Republic of China; 3 Biomedical Engineering Department, Ming Chuan University, Taoyuan City, 333 Taiwan

**Keywords:** Kidney function, Glomerular filtration rate, Exercise, Comorbidity, Risk factor

## Abstract

**Background:**

Impaired kidney function is the hallmark of chronic kidney disease (CKD), and is associated with increased risk of all-cause mortality in the elderly. In the present cross-sectional population-based study, we aimed to evaluate the associations between lifestyle factors (exercise habit, alcohol consumption, smoking history, and betel nut chewing) and decreased kidney function.

**Methods:**

The data from the Taipei City Elderly Health Examination Database (2006 to 2012) were extracted. Associations between risk factors and reduced estimated Glomerular filtration rate (eGFR) were evaluated by regression and stratification analyses.

**Results:**

A total of 297,603 participants were included in the final analysis, and 29.7% of them had reduced eGFR. Smoking was significantly associated with an elevated risk of reduced eGFR. While, physical exercise conferred to a significantly decreased adjusted odds ratio (aOR) in reduced eGFR (regular exercise, aOR = 0.79; occasional exercise, aOR = 0.87). Furthermore, the protective effect of exercise habit against reduced eGFR was not affected by comorbid conditions, such as hypertension, diabetes, obesity, and cardiovascular disease.

**Conclusions:**

Engaging in physical exercise was beneficially associated with reduced eGFR in older individuals. Longitudinal or prospective studies are warranted for confirmation and extrapolation of the current findings.

## Background

Chronic kidney disease (CKD) is a debilitating disease that affects around 11–13% of the global population and is estimated prevalent in 1.5 million individuals in Taiwan [1, 2]. A Korean National Health and Nutrition Evaluation Survey study indicated that the CKD prevalence rates were 3.5 and 2.4% in male and female adults, respectively, in 2013 in South Korea [3]. In addition, among European general populations, the CKD prevalence varied from 3.31% in Norway to 17.3% in northeast Germany [4]. Furthermore, a systematic review and meta-analysis of observational studies concluded that in general populations, the CKD prevalence rates were 3.5, 3.9, 7.6, 0.4, and 0.1% for CKD Stage 1, Stage 2, Stage 3, Stage 4, and Stage 5, respectively [1]. However, according to a large-scale National Health and Nutrition Evaluation Survey study in the US, the CKD prevalence is 46.8% in elderly people aged over 70 years, which is much higher than that in younger individuals [5]. CKD is diagnosed by the presence of abnormalities in estimated glomerular filtration rate (eGFR) and markers of kidney damage that include albuminuria and hematuria for more than 3 months [6]. A person with eGFR < 60 ml/min/1.73m^2^ is said to have impaired kidney function. It has been shown that lower eGFR and albuminuria are associated with higher prevalence and incidence of kidney failure, cardiovascular disease (CVD), CVD-associated mortality, and all-cause mortality in the elderly [7–11]. Alternatively, CVD itself and its risk factors, including diabetes, obesity and hypertension, are associated with the incidence and progression of CKD [12].

Physical activity and exercise have been shown to improve the quality of life and reduce the risk of dyslipidemia and CVD in the elderly [13–15]. A good physical performance is associated with reduction in all-cause mortality among older individuals with CKD [16]. Nevertheless, the benefits of physical exercise in preventing CKD or impaired kidney function are under debate. A cross-sectional study showed that higher levels of physical activity and lower levels of sedentary behavior were associated with reduced risk for lower eGFR [17]. However, the Framingham Heart Study found no significant association between physical activity and incident eGFR < 60 or rapid eGFR decline [18].

As the world population is aging, it is estimated that over 1.6 billion people worldwide will be 65 years and older by 2050, projecting an increasing burden on the health care system and society [19]. Given the unfavorable outcomes and increased prevalence of comorbidities in older people with CKD, primary care and preventive lifestyle measures are both important aspects in maintaining kidney health in these individuals [20]. Herein, we hypothesized that lifestyle factors may affect the risk for reduced eGFR in adults aged 65 or over. To examine this hypothesis, we conducted this cross-sectional population-based study to evaluate the associations between reduced eGFR and various lifestyle factors, including exercise habit, alcohol consumption, smoking history, and betel nut chewing, in a community-dwelling elderly population in Taipei, Taiwan.

## Methods

### Data source and study population

The present study analyzed the data derived from the Taipei City Elderly Health Examination Database (2006 to 2012), which collected health examination data from recruited community-dwelling Taipei citizens aged 65 years or older. The Taipei City Elderly Health Examination Database is sponsored by the Department of Health, Taipei City Government, and has been used for the purpose of research as previously described [13, 21–23]. In the present cross-sectional study, participants who had missing values for age, gender, or the serum creatinine level were excluded, because all these variables were required for the calculation of eGFR based on the Chronic Kidney Disease Epidemiology Collaboration (CKD-EPI) formula [24]. In addition, participants who had renal cancer, kidney disease, nephrectomy, kidney transplantation, and renal failure were also excluded.

### Ethical considerations

The present cross-sectional population-based study was approved by the institutional review board of the Taipei City Hospital (TCHIRB-10514118-W), and informed consent was waived due to the retrospective nature of this study. All participants of the Taipei City Elderly Health Examination Database provided written informed consent at the time of health examination, authorizing the Taipei City Government access to their data for research purposes. All personal identifying information had been delinked in the Taipei City Elderly Health Examination Database.

### Data extraction

The participants’ cross-sectional demographic and socioeconomic characteristics, lifestyle factors, blood and urine variables, and comorbidity were extracted from the Taipei City Elderly Health Examination Database. The demographic and socioeconomic characteristics included age, gender, marital status, body mass index (BMI), highest education degree, and income level. Lifestyle factors consisted of exercise habit, alcohol consumption, smoking history, and betel nut chewing. Blood and urine variables were the levels of fasting glucose, total cholesterol, triglyceride, high density lipoprotein (HDL), urine acid, blood urea nitrogen (BUN), creatinine, and proteinuria. Finally, the comorbidities such as hypertension, diabetes mellitus, dyslipidemia, hyperuricemia, urinary tract stones, CVD, and cancer were analyzed.

### Statistical analysis

Descriptive statistics of the participants’ demographic characteristics and blood and urine variables were presented as number (n) and percentage (%) or mean ± standard deviation (SD). Differences in categorical variables were tested by Pearson’s chi-square test, and those in numerical variables by two-sample t-test. Logistic regression analyses were performed to identify the risk factors associated with decreased kidney function (defined as eGFR < 60 ml/min/1.73m^2^ in the present study). Multivariate logistic regression analysis was performed in the total population after adjusting for all variables that were significant in the univariate regression model. Furthermore, participants were further stratified based on gender, hypertension, diabetes, obesity, and CVD to assess the possible confounding effect of gender, hypertension, diabetes, obesity, or CVD on association between exercise habit and reduced kidney function. In the stratified analysis, multivariate model was adjusted for other significant variables identified in the univariate regression model in the total population. All statistical analyses were two-sided and a *p* value < 0.05 was considered statistically significant. All statistical analyses were performed using SAS software version 9.4 (SAS Institute Inc., Cary, NC, USA).

## Results

**Fig. 1:**
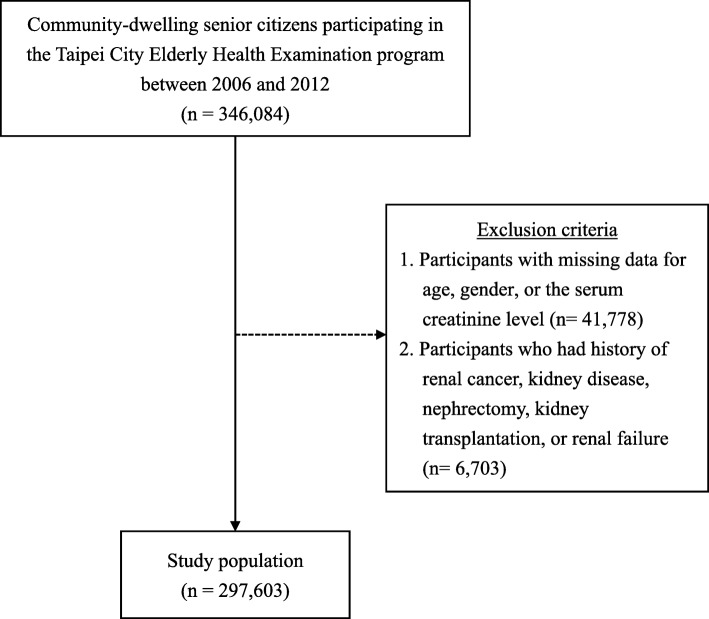
Flow chart of the study population

The present cross-sectional study accessed the data derived from 346,084 community-dwelling individuals aged 65 and over who participated in the Taipei City Elderly Health Examination program between 2006 and 2012. Altogether 41,778 participants who missing data for age, gender, or the serum creatinine level were excluded. In addition, 6703 participants who had history of renal cancer, kidney disease, nephrectomy, kidney transplantation, or renal failure were also excluded. As a result, a total of 297,603 participants were included in the final analysis (Fig. 1).

Demographic, socioeconomic, lifestyle factors, blood and urine variables, and comorbidities of the total population are presented in Table 1. The mean age of all participants was 75 years, and 51.5% of them were male. The majority of participants were married/cohabiting (73.8%), and 46.1% of participants had normal BMI. Regarding socioeconomic characteristics, 49.8% had high school diploma or higher, and 95.7% were financially better than poor. Most participants had regular exercise habit (49.6%), no drinking habit (97.6%), no current smoking habit (93.0%), and no betel-nut chewing habit (99.1%). In the present cohort, dyslipidemia (66.3%), hypertension (43.5%), CVD (20.8%), hyperuricemia/gout (19.9%), and diabetes (17.5%) were present as comorbidities, and 8.1% of participants had proteinuria (Table 1).

**Table 1:** Demographic, socioeconomic, and lifestyle factors of the total study population

Variables	Total^a^ (*n* = 297,603)
**Demographics**	
Age	75.0 ± 6.7
Gender	
Female	144,260 (48.5)
Male	153,343 (51.5)
Marital status	
Married/cohabiting	219,615 (73.8)
Widowed/divorced/separated	62,041 (20.9)
Never married	14,277 (4.8)
Missing value	1670 (0.5)
BMI^b^	
Underweight	11,484 (3.9)
Normal	137,246 (46.1)
Overweight	93,162 (31.3)
Obese	53,068 (17.8)
Missing value	2643 (0.9)
**Socioeconomic factors**	
Educational attainment	
With high school diploma or higher degree	148,298 (49.8)
Without high school diploma	141,261 (47.5)
Missing value	8044 (2.7)
Income level	
Not poor	284,808 (95.7)
Poor	12,795 (4.3)
**Lifestyle factors**	
Exercise habit	
No	33,021 (11.1)
Occasional	93,125 (31.3)
Regular	147,554 (49.6)
Missing value	23,903 (8.0)
Alcohol drinking	
No	290,524 (97.6)
Yes	6052 (2.0)
Missing value	1027 (0.4)
Current smoking	
No	276,610 (93.0)
Yes	20,047 (6.7)
Missing value	946 (0.3)
Betel nut chewing	
No	294,898 (99.1)
Yes	1041 (0.4)
Missing value	1664 (0.5)
**Blood and urine variables**	
Fasting glucose	104.7 ± 24.4
Total cholesterol	194.4 ± 35.2
Triglyceride	120.7 ± 72.9
HDL	54.6 ± 14.9
Uric acid	5.9 ± 1.6
BUN	17.6 ± 5.8
Creatinine	1.0 ± 0.4
Proteinuria	24,165 (8.1)
**Comorbidity**^**c**^	
Hypertension	129,492 (43.5)
Diabetes	52,186 (17.5)
Dyslipidemia	197,373 (66.3)
Hyperuricemia/gout	59,201 (19.9)
Urinary tract stones	6464 (2.2)
Cardiovascular disease	61,895 (20.8)
Cancer	1558 (0.5)

^a^ Data of the total study population were presented as mean ± standard deviation or number of participants (% of total cohort)

^b^ Underweight: BMI < 18.5; Normal: 8.5 ≤ BMI < 24; Overweight: 24 ≤ BMI < 27; Obese: BMI ≥ 27

^c^ Some participants had multiple comorbidities

Abbreviation: *BMI* body mass index; *BUN* blood urea nitrogen; *HDL* high density lipoprotein

Included participants were then divided into two groups based on eGFR variables: the reduced eGFR group (participants with eGFR < 60 ml/min/1.73m^2^; *n* = 88,219, 29.7%) and the normal eGFR group (participants with eGFR ≥60 ml/min/1.73m^2^; *n* = 209,384, 70.3%). Demographic, socioeconomic, lifestyle factors, blood and urine variables, and comorbidities of the two groups are shown in Table 2. Almost all demographic, socioeconomic, and lifestyle factors including physical exercise were significantly different between two eGFR groups, except for alcohol drinking (*p* = 0.1268) (Table 2). The reduced eGFR group had an older mean age and were more often male than the normal eGFR group. Compared with participants with normal eGFR, participants with decreased kidney function had slightly different fasting glucose, total cholesterol, triglyceride, and HDL levels, but these differences were small and of little clinical significance. In contrast, the differences in uric acid, BUN, creatinine, and percentage of participants with proteinuria were more pronounced in the two groups and were statistically significant. For comorbidities, significantly higher percentages of hypertension (53.9%), diabetes (22.5%), dyslipidemia (68.5%), hyperuricemia/gout (36.5%), and CVD (27.3%) cases were observed in the reduced eGFR group compared to those of the normal eGFR group (Table 2).

**Table 2:** Demographic, socioeconomic, and lifestyle factors of participants with decreased or normal eGFR

Variables	eGFR (ml/min/1.73m^2^)		
	Reduced (< 60) *n* = 88,219	Normal (≥ 60) *n* = 209,384	*p*-value
**Demographics**			
Age	77.7 ± 6.7	73.9 ± 6.3	< 0.001
Gender			< 0.001
Female	35,035 (39.7)	109,225 (52.2)	
Male	53,184 (60.3)	100,159 (47.8)	
Marital status			< 0.001
Married/cohabiting	62,820 (71.2)	156,795 (74.9)	
Widowed/divorced/separated	19,878 (22.5)	42,163 (20.1)	
Never married	4812 (5.5)	9465 (4.5)	
Missing value	709 (0.8)	961 (0.5)	
BMI ^a^			< 0.001
Underweight	2762 (3.1)	8722 (4.1)	
Normal	36,575 (41.5)	100,671 (48.1)	
Overweight	29,309 (33.2)	63,853 (30.5)	
Obese	18,568 (21.1)	34,500 (16.5)	
Missing value	1005 (1.1)	1638 (0.8)	
**Socioeconomic factors**			
Educational attainment			< 0.001
With high school diploma or higher degree	43,684 (49.5)	104,614 (50.0)	
Without high school diploma	41,575 (47.1)	99,686 (47.6)	
Missing value	2960 (3.4)	5084 (2.4)	
Income level			< 0.001
Not poor	83,260 (94.4)	201,548 (96.3)	
Poor	4959 (5.6)	7836 (3.7)	
**Lifestyle factors**			
Exercise habit			< 0.001
No	12,212 (13.8)	20,809 (9.9)	
Occasional	28,282 (32.1)	64,843 (31.0)	
Regular	41,105 (46.6)	106,449 (50.8)	
Missing value	6620 (7.5)	17,283 (8.3)	
Alcohol drinking			0.1268
No	86,177 (97.7)	204,347 (97.6)	
Yes	1727 (2.0)	4325 (2.1)	
Missing value	315 (0.3)	712 (0.3)	
Current smoking			< 0.001
No	81,224 (92.1)	195,386 (93.3)	
Yes	6709 (7.6)	13,338 (6.4)	
Missing value	286 (0.3)	660 (0.2)	
Betel nut chewing			0.0236
No	87,470 (99.2)	207,428 (99.1)	
Yes	270 (0.3)	771 (0.4)	
Missing value	448 (0.5)	1185 (0.5)	
**Blood and urine variables**			
Fasting glucose	106.9 ± 27.6	103.8 ± 22.9	< 0.001
Total cholesterol	191.9 ± 36.1	195.4 ± 34.8	< 0.001
Triglyceride	130.8 ± 77.9	116.5 ± 70.3	< 0.001
HDL	52.0 ± 14.6	55.7 ± 14.8	< 0.001
Uric acid	6.6 ± 1.7	5.6 ± 1.4	< 0.001
BUN	21.3 ± 7.7	16.0 ± 3.9	< 0.001
Creatinine	1.3 ± 0.5	0.9 ± 0.2	< 0.001
Proteinuria	13,758 (15.6)	10,407 (5.0)	< 0.001
**Comorbidity**			
Hypertension	47,553 (53.9)	81,939 (39.1)	< 0.001
Diabetes	19,802 (22.5)	32,384 (15.5)	< 0.001
Dyslipidemia	60,389 (68.5)	136,984 (65.4)	< 0.001
Hyperuricemia/gout	32,220 (36.5)	26,981 (12.9)	< 0.001
Urinary tract stones	1887 (2.1)	4577 (2.2)	0.4224
Cardiovascular disease	24,101 (27.3)	37,794 (18.1)	< 0.001
Cancer	478 (0.5)	1080 (0.5)	0.3688

Data were presented as mean ± standard deviation or number of participants (% of variables)

^a^ Underweight: BMI < 18.5; Normal: 8.5 ≤ BMI < 24; Overweight: 24 ≤ BMI < 27; Obese: BMI ≥ 27

Abbreviations: *BMI* body mass index; *eGFR* estimated glomerular filtration rate

The associations between decreased kidney function and demographic and socioeconomic characteristics, lifestyle factors, proteinuria, and comorbidity in the total study population were evaluated using logistic regression analyses (Table 3). The variables that were significant in univariate logistic regression analysis were included in the multivariate logistic regression model. The results of multivariate analysis revealed that participants who had either regular or occasional exercise habit consistently showed significantly lower odds of having decreased kidney function than those who did not exercise (occasional: adjusted OR [aOR] = 0.87, 95% CI = 0.84–0.90; regular: aOR = 0.79, 95% CI = 0.77–0.81). Betel nut-chewing was associated with lower risk of prevalent CKD in the univariate logistic regression model, but not after multivariate adjustment. In contrast, older age (aOR = 1.08, 95% CI = 1.08–1.09), and the status of never married (aOR = 1.10, 95% CI = 1.05–1.14), current smoker (aOR = 1.15, 95% CI = 1.11–1.19), proteinuria (aOR = 2.56, 95% CI = 2.49–2.64), dyslipidemia (aOR = 1.14, 95% CI = 1.12–1.16), or hyperuricemia/gout (aOR = 3.39, 95% CI = 3.32–3.46) were significantly associated with higher odds of having decreased kidney function. Furthermore, well-known indicators of CKD such as male gender (aOR = 1.36, 95% CI = 1.34–1.39), overweight/obesity (overweight: aOR = 1.14, 95% CI = 1.12–1.16; obese: aOR = 1.16, 95% CI = 1.13–1.19; compared with reference group), hypertension (aOR = 1.33, 95% CI = 1.31–1.35), diabetes (aOR = 1.26, 95% CI = 1.23–1.29), and cardiovascular disease (aOR = 1.25, 95% CI = 1.22–1.27) also showed higher odds of having decreased kidney function than those without the corresponding comorbidities (Table 3).

**Table 3:** Associations between study variables and decreased kidney function (eGFR < 60) in the total study population

	OR^a^ (95% CI)	aOR^b^ (95% CI)
**Demographics**		
Age	1.09 (1.09–1.09)*	1.08 (1.08–1.09)*
Gender		
Female	Ref.	Ref.
Male	1.66 (1.63–1.68)*	1.36 (1.34–1.39)*
Marital status		
Married/cohabiting	Ref.	Ref.
Widowed/divorced/separated	1.18 (1.15–1.20)*	1.02 (0.99–1.04)
Never married	1.27 (1.22–1.32)*	1.10 (1.05–1.14)*
BMI^c^		
Normal weight	Ref.	Ref.
Underweight	1.15 (1.10–1.20)*	0.78 (0.74–0.82)*
Overweight	1.45 (1.39–1.52)*	1.14 (1.12–1.16)*
Obese	1.70 (1.62–1.78)*	1.16 (1.13–1.19)*
**Socioeconomic factors**		
Educational attainment		
With high school diploma or higher degree	Ref.	
Without high school diploma school	1.0 (0.98–1.01)	
Income level		
Not poor	Ref.	Ref.
Poor	1.53 (1.48–1.59)*	1.03 (0.98–1.07)
**Lifestyle factors**		
Exercise habit		
No	Ref.	Ref.
Occasional	0.74 (0.72–0.76)*	0.87 (0.84–0.90)*
Regular	0.66 (0.64–0.67)*	0.79 (0.77–0.81)*
Alcohol drinking (vs. no)	0.95 (0.90–1.01)	
Current smoking (vs. no)	1.21 (1.17–1.25)*	1.15 (1.11–1.19)*
Betel nut chewing (vs. no)	0.83 (0.72–0.95)*	0.86 (0.73–1.00)
**Blood and urine variables**		
Proteinuria	3.53 (3.44–3.63)*	2.56 (2.49–2.64)*
**Comorbidity**		
Hypertension	1.82 (1.79–1.85)*	1.33 (1.31–1.35)*
Diabetes	1.58 (1.55–1.61)*	1.26 (1.23–1.29)*
Dyslipidemia	1.15 (1.13–1.17)*	1.14 (1.12–1.16)*
Hyperuricemia/gout	3.89 (3.82–3.96)*	3.39 (3.32–3.46)*
Urinary tract stones	0.98 (0.93–1.03)	
Cardiovascular disease	1.71 (1.68–1.74)*	1.25 (1.22–1.27)*
Cancer	1.05 (0.94–1.17)	

Note: Star (*) indicates statistical significance (*p* < 0.05)

^a^ Univariate regression analysis

^b^ Multivariate regression analysis

^c^ Underweight: BMI < 18.5; Normal: 8.5 ≤ BMI < 24; Overweight: 24 ≤ BMI < 27; Obese: BMI ≥ 27

Abbreviation: *aOR* adjusted OR; *BMI* body mass index; *CI* confidence interval; *OR* odds ration; *Ref* reference

To examine whether gender, hypertension, diabetes, obesity, and cardiovascular disease affect the association between exercise habit and decreased kidney function, stratified analyses were performed (Table 4). The protective effect of exercise habit on the odds of decreased kidney function was observed in both male and female participants (female: aOR = 0.80, 95% CI = 0.77–0.83; male: aOR = 0.85, 95% CI = 0.81–0.88), as determined by multivariate model after adjusting for age, marital status, current smoker, proteinuria, dyslipidemia and hyperuricemia/gout. The exercise habit was associated with significantly lower odds of decreased kidney function in participants with or without hypertension (hypertension: aOR = 0.87, 95% CI = 0.84–0.90; without hypertension: aOR = 0.84, 95% CI = 0.81–0.87), with or without diabetes (diabetes: aOR = 0.83, 95% CI = 0.78–0.87; without diabetes: aOR = 0.87, 95% CI = 0.84–0.89), with or without obesity (obesity: aOR = 0.82, 95% CI = 0.78–0.87; without obesity: aOR = 0.85, 95% CI = 0.82–0.88), and with or without CVD (CVD: aOR = 0.87, 95% CI = 0.83–0.92; without CVD: aOR = 0.85, 95% CI = 0.83–0.88) (Table 4).

**Table 4:** Effects of gender, diabetes, hypertension, obesity and cardiovascular disease on the association between exercise habit and decreased kidney function, determined by stratified analyses

Stratification	The association between exercise habit ^a^ and decreased eGFR (< 60)	
	OR (95% CI)	aOR (95% CI)
**Gender**		
Female	0.60 (0.58–0.62)*	0.80 (0.77–0.83)*
Male	0.70 (0.68–0.73)*	0.85 (0.81–0.88)*
**Hypertension**		
Yes	0.72 (0.69–0.74)*	0.87 (0.84–0.90)*
No	0.68 (0.66–0.71)*	0.84 (0.81–0.87)*
**Diabetes**		
Yes	0.66 (0.62–0.69)*	0.83 (0.78–0.87)*
No	0.72 (0.70–0.74)*	0.87 (0.84–0.89)*
**Obesity**^**b**^		
Yes	0.73 (0.69–0.77)*	0.82 (0.78–0.87)*
No	0.69 (0.67–0.71)*	0.85 (0.82–0.88)*
**Cardiovascular disease**		
Yes	0.70 (0.67–0.74)*	0.87 (0.83–0.92)*
No	0.71 (0.69–0.73)*	0.85 (0.83–0.88)*

Note: Star (*) indicates statistical significance (*p* < 0.05)

Multivariate model was adjusted for age, marital status, current smoker, proteinuria, dyslipidemia and hyperuricemia/gout

^a^ Exercise habit was defined as with regular or occasional exercise

^b^ Obesity was defined as BMI ≥ 27

Abbreviation: *aOR* adjusted OR; *CI* confidence interval; *eGFR* estimated glomerular filtration rate; *OR* odds ratio

## Discussions

To the best of our knowledge, this is the largest cross-sectional study involving 297,603 participants representing a generalized community-dwelling elderly population, which investigated the associations between lifestyle factors and reduced kidney function. The present study found that smoking was associated with increased odds of an impaired eGFR, while physical exercise was favorable for kidney function. Comorbid conditions including CVD, hypertension, obesity, and diabetes that are known to contribute to risk of developing CKD were also found associated with reduced eGFR in the present study. The reduced odds of having a decreased eGFR conferred by physical exercise habit in the presence of different comorbid conditions associated with CKD was further demonstrated by stratification analysis, where the benefit of physical exercise was consistently shown.

In line with the decreased odds of impaired kidney function associated with physical exercise observed by us, the Maastricht Study found higher physical activity beneficially associated with kidney function [25]. This prospective observational population-based study recruited individuals aged between 40 and 75 years through public engagement, and further reported that sedentary behavior was associated with adverse kidney function [25]. Similarly, a cross-sectional study involving 1350 men with a mean age of 78.5 years showed that higher physical activity and a less sedentary behavior were associated with favorable kidney function [17], in which the level of physical activity and sedentary behavior were measured objectively over a 7-day period by wearable physical activity monitors. Although data concerning physical exercise habit and other lifestyle factors were collected via a self-administered questionnaire in our study, the study by Parson et al. (2017) [17] strengthens our observation that both participant groups with occasional or regular exercise habit showed reduced odds of eGFR < 60 compared with those who did not exercise. Furthermore, a meta-analysis research indicated that physical activity significantly reduces blood pressure in patients who are aged 50 and older and had chronic renal failure [26].

On the other hand, the Framingham Heart Study did not observe associations between baseline physical activity and incident impaired eGFR or rapid eGFR decline over a median follow-up of 6.6 years in older adults, who were offspring of the original Framingham cohort [18]. In addition, a pilot randomized controlled study tested the preventive benefits of exercise training on cardiovascular, vascular and renal health in adults with stage 3–4 CKD found that the 1-year exercise intervention did not slow the progression of kidney disease [27]. It should be noted that the two studies described above were prospective and longitudinal studies, and involved different age groups compared with the present study. Nevertheless, the disparity in findings stresses the need of further research to define the preventive role of active physical exercise in kidney health.

Supportive of our findings, previous studies reported smoking and betel-nut chewing as hazardous lifestyle factors with a negative impact on the risk of developing CKD [28, 29]. In the present study, we further illustrate that these lifestyle habits are also unfavorable in people > 65 years-old. On the other hand, despite the Korean National Health and Nutrition Examination Survey (KNHANES) showing negative correlation between alcohol drinking and renal dysfunction especially in men [30], this was not observed in the present study. Discrepancy results may be in part due to cultural differences in drinking, as around 76.5% of participants in the KNHANES study were moderate to heavy drinkers, in contrast to our cohort, where only 2% were alcohol drinkers. The other discrepancy was the age of participants, as we focused on elderly individuals > 65 years-old while Kim et al. (2014) recruited adults aged 20 years and over [30].

One of limitations of the present study is the lack of longitudinal observations due to the cross-sectional nature of the study design. In addition, physical exercise was not objectively measured, and diet quality that may correlate with physical activity was not assessed. Furthermore, the participants with reduced eGFR are likely to be sicker and therefore are less likely to engage in exercise, so the possibility of reverse causality between reduced eGFR and physical activity engagement cannot not be ruled out. However, our findings based on a large cohort data extracted from the Taipei City Elderly Health Examination Database should represent the generalized elderly population. Further studies adopting a longitudinal design or prospective enrollment of elderly participants with objective physical exercise measurements may assist to confirm the results of the present study.

## Conclusions

The present study found that physical exercise, among other favorable lifestyle habits, is beneficially associated with eGFR in a large cohort of community-dwelling elderly people aged > 65 years. The reduced odds in having an eGFR < 60 was significantly associated with occasional or regular physical in the presence or absence of CVD, hypertension, obesity, and diabetes, which are known to increase the risk of CKD. Future studies utilizing either longitudinal observation, prospective design, or active measurement of physical exercise levels should confirm our current findings of kidney function in the elderly and assist strategic planning of relevant public health policy.

## Data Availability

The data analyzed for the current study are available from the corresponding author on reasonable request.

## Abbreviations

aOR: Adjusted odds ratio
BMI: Body mass index
BUN: Blood urea nitrogen
CKD: Chronic kidney disease
CKD-EPI: Chronic Kidney Disease Epidemiology Collaboration
CVD: Cardiovascular disease
eGFR: Estimated Glomerular filtration rate
HDL: High-density lipoprotein
KNHANES: Korean National Health and Nutrition Examination Survey
SD: Standard deviation
